# Changing the Approach to Anticoagulant Therapy in Older Patients with Multimorbidity Using a Precision Medicine Approach

**DOI:** 10.3390/ijerph15081634

**Published:** 2018-08-02

**Authors:** Angela Koverech, Valeriano Soldati, Vittoria Polidori, Leda Marina Pomes, Luana Lionetto, Matilde Capi, Andrea Negro, Maurizio Simmaco, Paolo Martelletti

**Affiliations:** 1 Department of Clinical and Molecular Medicine, Azienda Ospedaliera Universitaria S. Andrea, via di Grottarossa 1035/1039, 00189 Rome, Italy; angela.koverech@uniroma1.it (A.K.); andrea.negro@uniroma1.it (A.N.); 2 Department of Physiology and Pharmacology, Sapienza University of Rome, 00185 Roma, Italy; 3 NESMOS Department, S. Andrea Hospital, University of Rome Sapienza, 00185 Rome, Italy; vsoldati@ospedalesantandrea.it (V.S.); vittoria.polidori@ospedalesantandrea.it (V.P.); maurizio.simmaco@uniroma1.it (M.S.); 4 Residency Program in Laboratory Medicine, Gabriele d’Annunzio University, 66100 Chieti, Italy; ledama@hotmail.it; 5 Advanced Molecular Diagnostics Unit, IDI-IRCCS, 00168 Rome, Italy; luanalionetto@gmail.com; 6 Laboratory of Clinical Chemistry, Sant’Andrea Hospital, via di Grottarossa 1035/1039, 00189 Rome, Italy; matildecapi@gmail.com

**Keywords:** ageing, multimorbid patients, polytherapy, precision medicine, enzymes, polymorphisms

## Abstract

The ageing of the world population has resulted in an increase in the number of older patients with multimorbid conditions receiving multiple therapies. This emerging clinical scenario poses new challenges, which are mostly related to the increased incidence of adverse effects. This translates into poor clinical care, reduced cost-effectiveness of drug therapies, and social isolation of multimorbid patients due to reduced autonomy. A strategy to address these emerging challenges could involve the personalization of therapies based on the clinical, molecular, and genetic characterization of multimorbid patients. Anticoagulation therapy is a feasible model to implement personalized medicine since it generally involves older multimorbid patients receiving multiple drugs. In this study, in patients with atrial fibrillation, the use of the new generation of anticoagulation therapy, i.e., direct oral anti-coagulants (DOACs), is based on a preliminary assessment of the molecular targets of DOACS and any possible drug–drug interactions. Then, the genetic polymorphism of enzymes metabolizing DOACs is studied. After DOAC prescription, its circulating levels are measured. Clinical data are being collected to assess whether this personalized approach improves the safety and efficacy profiles of anticoagulation therapy using DOACs, thereby reducing the costs of healthcare for ageing multimorbid patients.

## 1. Introduction

The populations of the majority of countries throughout the world are progressively ageing. A clear example is given by the demographic changes which have occurred in Italy over the last 60 years. In 1957, the median age of the Italian population was 30.6 years, which was below the median age of the six countries founding members of the European Union, i.e., 33 years [1]. After 60 years, Italy’s median age has increased by almost 50% and is now above the median age of the remaining 28 EU countries, i.e., 45.5 vs. 42.6 years, respectively [1]. At the global level, it has been recently reported that between 1990 and 2016, a profound shift toward deaths at older ages occurred, with a 178% increase in deaths in those aged 90–94 years and a 210% increase in deaths in those aged over 95 years [2].

The clinical, economic, and social implications of population ageing are manifold, but mostly related to the potential exponential increase of the prevalence of chronic diseases, yielding a significant and almost unaffordable increase in healthcare costs. In China, population growth and ageing has led to a steady increase in the ischemic heart disease burden [3]. In Germany, persons ageing in socially disadvantaged areas have a higher chance of developing chronic obstructive pulmonary disease (COPD), even when they are not necessarily directly affected by deprivation on an individual level [4]. About 6% of the Dutch population and about 25% of the people older than 65 years will suffer from diabetes in 2025 [5]. Relative to 2005, this is an increase of 70% in 20 years. In the UK, people born in the 1930s had a 37% chance of developing cancer during their lifetime, which increased to 50% for those born in the 1960s, mainly due to population ageing [6].

Another point of view highlighting the epidemiological shift which occurred in the last few decades involves the observation of the trends of the causes of death worldwide. In 2016, deaths from non-communicable diseases (NCDs) represented 72.3% of deaths, with 19.3% of deaths in that year caused by communicable, maternal, neonatal, and nutritional (CMNN) diseases and a further 8.4% as a result of injuries [2]. Of great interest is the evidence that although age-standardized rates of death from NCDs decreased globally between 2006 and 2016, total numbers of these deaths increased [2]. The interpretation of these data suggest that the current medical treatments extend survival of patients with chronic diseases who die at an older age, likely with disabilities. Also, since current medical treatments cannot cure patients, the number of persons with NCDs is increasing year after year.

The economic impact of population ageing is enormous. It is estimated that in 2030 the economic burden of cancer care will be approximately 460 billion USD/year, a more than 50% rise from the 2007 costs [7]. However, a recent analysis of healthcare costs in the Veneto region, Italy, showed that aging itself is not the main determinant of this increase, which is better explained by the accumulation of chronic conditions and the resulting multimorbidity [8]. Therefore, dissociating ageing from multimorbidity by implementing healthy lifestyle is an ambitious goal which would yield significant cost savings. Unfortunately, compliance to lifestyle changes is very poor and effective pro-successful ageing pharmacological approaches have not been developed thus far.

An area in need of improvement in the management of multimorbid older adults is the safety and efficacy of drug therapies. Many studies have clearly shown that many drugs are less effective in the real world than in the protected environment of clinical trials. Davis et al. showed that among 68 cancer drug indications approved by the European Medicines Agency (EMA) in the period 2009–2013, and with a median of 5.4 years of follow up, only 35 (51%) were associated with a significant improvement in survival or quality of life over alternative treatment options, placebo, or add-on treatments [9]. A potential explanation for the reduced efficacy of medical treatments in daily practice could be related to the mismatch between the mean age of patients enrolled in clinical trials and that of hospitalized or ambulatory patients. In general, patients older than 65 years are not enrolled in clinical trials, whereas a large proportion of the hospitalized and ambulatory patients are older than 70 years. Indeed, Stuntz et al. reported that older age is associated with increased likelihood of hospital admission in the USA [10].

It should be also noted that multimorbid older adults are almost invariably prescribed a high number of drugs. A recent Brazilian survey showed that prevalence of at least one chronic-use medicine among older adults was 93.0% [11]. Of the total number of older adults, 18.0% used at least five medications [11]. Polypharmacy was higher among the oldest individuals (20.0%), in those with poor self-perceived health (35.0%), in obese individuals (26.0%), in those with reported health insurance (23.0%) or hospitalization in the previous year (31.0%), and among those who reported diabetes (36.0%) and heart diseases (43.0%) [11]. Polypharmacy in older adults is associated with higher likelihood of adverse effects. Rausch et al. showed that at a population level, the number of different dispensed medications starting from three or more remains associated with adverse drug events even after adjusting for known inappropriate drug uses in older adults [12]. Clinicians and patients need to be made aware of the increased likelihood of serious adverse drug events, not only in the case of documented inappropriate medications but also in the case of an increasing number of medications.

Consequently, to reduce the incidence of adverse events and enhance the efficacy of therapies in multimorbid older adults receiving polypharmacy, deprescribing should be implemented or a more thorough approach to the metabolism of drugs in older adults should be considered.

## 2. Anticoagulant Therapy: A Paradigm for Improving Outcome by Precision Medicine

Ageing is associated with the development of chronic diseases and their complications. Ageing plays a role in the genesis of atrial fibrillation [13], which is the most common type of arrhythmia diagnosed in clinical practice. The consequences of atrial fibrillation have been clearly established in multiple large observational cohort studies and include increased stroke and systemic embolism rates if no oral anticoagulation is prescribed, with increased morbidity and mortality [14]. With the worldwide aging of the population characterized by a large influx of “baby boomers” with or without risk factors for developing atrial fibrillation, an epidemic is forecasted within the next 10 to 20 years [14]. Although not all studies support this evidence, it is clear that atrial fibrillation is on the rise and significant of health resources are being invested in detecting and managing atrial fibrillation [14].

To prevent stroke and systemic embolism, oral anticoagulants are largely prescribed. However, most of the patients with atrial fibrillation are older adults and present with other chronic conditions. Multimorbidity is now becoming a major concern for the effective and safe treatment of patients with atrial fibrillation and in need of anticoagulation. A clear example is given by patients with chronic kidney disease and receiving oral anticoagulation. Kumar et al. assessed the association between anticoagulation, ischemic stroke, gastrointestinal and cerebral hemorrhage, and all-cause mortality in this specific clinical setting [15]. The study included 6977 patients with chronic kidney disease (i.e., estimated glomerular filtration rate of <50 mL/min/1.73 m^2^) and newly diagnosed atrial fibrillation. Of these, 2434 were on anticoagulants within 60 days of diagnosis and 4543 were not. The crude rates for ischemic stroke and hemorrhage were 4.6 and 1.2 after taking anticoagulants and 1.5 and 0.4 in patients who were not taking anticoagulant per 100 person years, respectively. The hazard ratios for ischemic stroke, hemorrhage, and all-cause mortality for those on anticoagulants were 2.60, 2.42, and 0.82, respectively compared with those who received no anticoagulation. Therefore, giving anticoagulants to older people with concomitant atrial fibrillation and chronic kidney disease is associated with an increased rate of ischemic stroke and hemorrhage but a paradoxical lowered rate of all-cause mortality [15].

For decades, warfarin, which inhibits vitamin K metabolism, represented the first choice for oral anticoagulation in patients with atrial fibrillation. The disadvantage of warfarin-based therapy is related to the need for regular monitoring of coagulation, indicating that under- or over-dosage are significant possibilities in daily clinical practice. More recently, new oral anticoagulants (NOAs), also known as direct oral anticoagulants (DOACs) have become available. They are now largely prescribed since it is not necessary to regularly monitor coagulation markers. Also, specific clinical advantages have been reported for the use of DOACs vs. warfarin. Bai et al. analyzed the effectiveness and safety of warfarin use compared with warfarin non-use and DOACs in atrial fibrillation patients aged ≥65 years [16]. After searching PubMed and the Cochrane Library, 26 studies were included, with 10 comparing warfarin with warfarin non-use and 16 comparing warfarin with DOACs [16]. The results showed that warfarin use was superior to no antithrombotic therapy and aspirin for stroke/thromboembolism prevention [16]. Warfarin use was associated with a non-significant increase in risk of major bleeding compared with no antithrombotic therapy and aspirin. DOACs were superior to warfarin for stroke/thromboembolism prevention, and also were associated with reduced risk of major bleeding.

Similarly, Inohara et al. showed that among 141,311 patients with intracerebral hemorrhage, 15,036 (10.6%) were taking warfarin, 4918 (3.5%) were taking DOACs preceding intracerebral hemorrhage, and 39,585 (28.0%) and 5783 (4.1%) were taking concomitant single and dual antiplatelet agents, respectively [17]. Patients with prior use of warfarin or DOACs were older and had higher prevalence of atrial fibrillation and prior stroke. Acute intracerebral hemorrhage stroke severity (measured by the National Institutes of Health Stroke Scale) was not significantly different across the three groups. The unadjusted in-hospital mortality rates were 32.6% for warfarin, 26.5% for DOACs, and 22.5% for no DOACs. Compared with patients with prior use of warfarin, patients with prior use of DOACs had a lower risk of in-hospital mortality [17].

This robust evidence of superior efficacy and safety should not lead us to overlook the inherent bleeding and thromboembolic risk of any anticoagulant therapy, including DOACs. Although it is acknowledged that inappropriate dosing of DOACs contributes to an increased risk of stroke [18], recent evidence shows that plasma concentrations of DOACs may significantly vary from patient to patient. Gulilat et al. reported that among 243 patients receiving DOACs (rivaroxaban, *n* = 94; apixaban, *n* = 149), a 60- and 50-fold interpatient variation in plasma concentration was observed for rivaroxaban and apixaban, respectively [19]. Approximately 12% of patients receiving rivaroxaban and 13% of patients receiving apixaban exceeded the 95th percentile for predicted maximum plasma concentration observed in clinical trials [19]. Therefore, DOAC plasma concentrations tend to be more variable than those observed in clinical trials, posing an additional clinical risk to the patients. Indeed, in a geriatric population, inter- and intra-individual coefficients of variation were 59.5% and 44.7% for peak and 74.5% and 44.6% for trough, respectively, and high through concentrations identified patients at risk of bleeding [20]. Identification of additional clinical and molecular determinants that more fully predict patients at risk for excessively high or low DOAC concentrations may enable a more precise DOAC dosing regimen for the individual patient.

It is acknowledged that drug–drug interactions are responsible of DOAC metabolism. In an in vitro series of experiments, Margelidon-Cozzolino et al. showed that phosphodiesterase type-5 inhibitors, a drug class commonly used in the treatment of pulmonary arterial hypertension, inhibit rivaroxaban and apixaban efflux in a cellular model of drug transport assay [21]. However, beyond drug–drug interactions which likely occur in patients with polypharmacy, the individual metabolic and genetic profile of older patients receiving DOACs may provide useful information to personalize treatments. In this regard, it is important to note that the translation into clinical practice of large randomized clinical trials may not yield the expected benefits to the older population, since this class of patients is frequently excluded from enrollment. Also, it should be noted that polymorbid patients are not usually considered for enrollment in clinical trials, whereas polymorbidity is highly prevalent among older adults treated in the real world.

## 3. A Hospital-Based Precision Medicine Project to Enhance the Efficacy and Effectiveness of Anticoagulation Therapy by DOACs

To minimize the inherent clinical risks associated with anticoagulant therapy, the different factors involved in the efficacy and effectiveness of DOACs should be preliminarily assessed. These are outlined in Table 1.

Clinical indications for anticoagulation therapy in general and for DOAC prescription in particular are informed by international guidelines, which have been adopted by national and regional health authorities. As an example, DOAC prescription in Italy is tightly controlled by the regional health systems, and divergences from the internationally acknowledged indications result in inappropriate prescriptions which are not reimbursed by the healthcare systems. This exposes the patient to unnecessary clinical risk and the prescribing doctor to litigation for malpractice in case of adverse events. It is therefore self-evident that the first step in personalizing anticoagulation therapy by DOACs requires careful medical history collection and clinical examination of the patients. Also, the clinical outcomes of older adult patients extend beyond classical metric parameters, since patient-centered outcomes and quality of life are of the utmost importance in informing prescription. However, matching disease with therapy is only an initial and general step in precision medicine. Indeed, likelihood of compliance to anticoagulant treatment within a regime of polypharmacy, as well the costs associated with complications and monitoring, play a role when considering adding or changing a medication. In our hospital, we are now implementing a protocol which aims at enhancing personalization of anticoagulation therapy by considering further factors involved in determining the adherence to DOAC-based therapy. This approach may reduce the clinical risk associated with anticoagulation therapy and ultimately save healthcare costs by reducing visits to emergency room or long-term consequences of adverse events on patients’ functional status and autonomy. This approach is being implemented within a general clinical framework, in which all the variables influencing adherence to treatment are considered (i.e., costs of monitoring and complications, life expectancy of the patients, impact of new medications on patient-centered outcomes and quality of life, etc.).

(1) Drug–drug interaction(s). As previously outlined, patients in need of anticoagulation therapy are frequently older, with polymorbidity, and receive polypharmacy. Consequently, after assessing the indication to anticoagulation, we collect precise information on the therapy the patient is already receiving. By using specific software (i.e., http://www.drugbank.ca/ or http ://bioinformatic.charite.de/trasformer), we then assess whether drug–drug interaction(s) already exists and which DOAC has the best metabolic profile so as to not interfere, or minimally interfere, with the existing metabolic and enzymatic homeostasis developed by the existing therapy. This analysis also includes assessment of potential interaction with P-glycoprotein.
(2) Circulating levels of the molecular target of DOACs. DOAC target activated factor X (factor Xa) or thrombin (factor IIa). It is well known and acknowledged that intra- and interindividual variability exist in coagulation parameters. Costongs et al. showed that intra-individual variations ranged from 0 to 6.6% and from 3.9 to 16.4% for three screening tests and six specific coagulation tests, respectively [22]. Also, critical differences varied from 9.9 to 19.5% and 14.8 to 46.7%, respectively [22]. We therefore included the measurement of factor Xa and factor IIa in the pre-treatment assessment of patients with clinical indication for DOAC-based anticoagulation therapy. This will help in identifying those patients with constitutional reduced levels of any of the targets of DOACs, and thus allow us to shift to the most appropriate prescription.
(3) Assessment of the expression/activity of enzymes involved in DOAC metabolism. The efficacy of DOAC-based therapy is influenced not only by drug–drug interactions and baseline levels of the molecular target, but also by the genetic polymorphisms of the enzymes involved in their metabolism, which results in their differential expression and activity. We therefore identified a panel of enzymes which metabolizes DOACs and we assessed the genes for each patient. In particular, we focus our attention on CYP450 (1A2, 2C8, 2C19, 3A4, and 3A5) For most of such genes, polymorphisms influencing their expression and activity are known. When the whole panel is received by the clinician with an accompanying letter by the clinical pathologist, it is easier to identify those patients who require a closer follow-up due to anticipated variability in the efficacy of DOACs.
(4) Measurement of the circulating levels of DOACs (after 90 days of therapy). It has been clearly demonstrated that variability in the circulating levels of DOACs increases the risk of clinically relevant complications. Testa et al. studied 330 patients receiving DOACs therapy and reported that mean inter-individual variability expressed as overall coefficient variation values for any DOAC prescribed and taken by patients was lower at peak (CV = 46%) than at trough (CV = 63%) [23]. Mean intra-individual variability was 36.6% at trough and 34.0% at peak [23]. Correlation with creatinine clearance was poor for all DOAC drugs and only dabigatran, an inhibitor of factor IIa, showed a significant correlation at trough [23]. The importance of this study for our purposes is highlighted by the evidence that high DOAC inter-individual variability cannot be explained by renal function alone. The potential clinical consequences of such large variability are highlighted by a more recent study involving 565 consecutive patients with atrial fibrillation receiving DOACs [24]. The results show that thromboembolic events occurred in 10 patients (1.8%) who had baseline concentration–trough levels in the lowest class of drug levels [24]. The incidence of thromboembolic events among patients with DOAC concentration trough results in the lowest level class was 2.4%, while it was 0% in the remaining groups [24]. The patients with thrombotic complications also had a mean CHA_2_ DS_2−_ VASc score, a composite score to assess of the risk of thromboembolic complications, higher than that of the total patient population: 5.3 (95% confidence interval 4.3–6.3) vs. 3.0 (95% confidence interval 2.9–3.1).

Based on these compelling evidence, we included in our protocol the measurement of DOACs concentrations after 90 days of therapy to identify those patients at higher risk of complications and thus in need of dose adjustment. It is acknowledged that our clinical protocol has inherent limitations which are mainly related to the complexity of treating multimorbid older patients receiving polypharmacy. These include failure to identify patients unlikely to be compliant with therapy or with the follow-up monitoring. Nevertheless, we believe that ultimate goals of this approach for patients in need of anticoagulant therapy, i.e., enhanced safety, efficacy, and effectiveness versus the historical comparator, justify its implementation.

## 4. Conclusions

Modern medicine is now advancing together with the development and implementation in clinical practice of procedures and therapies resulting from molecular dissection of the pathogenesis of diseases. However, the use of these new therapies does not necessarily yield better results. As an example, the SHIVA trial comparing the survival of cancer patients receiving molecularly targeted therapies vs. standard of care could not prove any benefit of the use of targeted therapies [25]. We strongly believe that effective precision medicine should encompass not only a precise assessment of the molecular targets to be addressed by new drugs, but a thorough evaluation of the genotypic and phenotypic characteristics of the individual patient. We believe that by using this approach, more patients will benefit from often expensive therapies, enhancing the cost-effectiveness of medical treatments. Ultimately and after global implementation, this protocol could contribute to increased healthy life expectancy as well reduced disability-adjusted life years [26,27].

## Figures and Tables

**Table 1:** Factors involved in the efficacy and effectiveness of direct oral anticoagulant (DOAC) therapy.

Indication for anticoagulant therapy
Indication for DOAC therapy
Drug–drug interaction(s)
Circulating levels of the molecular target of DOAC therapy
Assessment of the expression/activity of enzymes involved in DOAC metabolism
Measurement of the circulating levels of DOACs (after 90 days of therapy)
